# Stone Trails Across the Cervical Planes: Extensive Bilateral Cervicothoracic Calcifications Mimicking Invasive Pathology

**DOI:** 10.7759/cureus.90308

**Published:** 2025-08-17

**Authors:** Humna Rashid, Hadia Sohail, Muhammad Younus, Saleh Khurshied, Syeda Hoorya Faatmaa

**Affiliations:** 1 Otolaryngology - Head and Neck Surgery, Pakistan Institute of Medical Sciences, Islamabad, PAK

**Keywords:** conservative approach, head and neck calcifications, head and neck mass, long term follow up, soft-tissue calcification

## Abstract

Benign soft tissue calcifications in the neck are uncommon and can pose significant diagnostic challenges due to their clinical and radiological resemblance to malignant lesions. It is crucial to distinguish between these entities in order to prevent unnecessary and aggressive interventions. We describe the case of a 38-year-old woman who had an anterior neck swelling that had been painful, firm, and irregular for four years and had no previous comorbidities. With no accompanying symptoms like dysphagia, dyspnea, or voice abnormalities, the mass had an abrupt onset and did not progress. A fixed, firm swelling that moved with deglutition and tongue protrusion was discovered during the clinical examination. Imaging revealed widespread, pleomorphic calcifications extending from the cervical to the upper thoracic area. These extended transfascially and transcompartmentally, with a modest mass effect on nearby structures. Ultrasound-guided FNAC was non-diagnostic, and a subsequent incisional biopsy revealed calcified and fibrous tissue without evidence of malignancy. Since there were no compressive symptoms and the histology was benign, the patient was treated conservatively and monitored closely. This case emphasizes how crucial it is to perform a thorough clinical, radiographic, and histological investigation in order to differentiate benign calcified neck masses from malignancies. Conservative management with close monitoring may be appropriate in selected cases without compressive or systemic signs.

## Introduction

In humans, calcium deposition is physiologically limited to mineralized tissues like cartilage, teeth, and bones [1]. However, under certain pathological conditions, calcium salts can abnormally accumulate in soft tissues, a phenomenon known as heterotopic calcification. These deposits are typically amorphous and can arise in a variety of contexts [2]. Heterotopic calcifications can be broadly divided into two groups: metastatic calcification, which involves normal tissue because of elevated serum calcium or phosphate levels and is frequently caused by metabolic disorders like hyperparathyroidism or chronic kidney disease, and dystrophic calcification, which occurs in previously injured or necrotic tissues despite normal serum calcium and phosphate levels [3].

Soft tissue calcifications are common in the head and neck area and are typically discovered incidentally when imaging tests are conducted for unrelated conditions. While most of these calcifications are asymptomatic and benign, their appearance can occasionally mimic more serious pathology, especially when extensive or atypically distributed. Common areas in the neck where calcifications may occur include the tonsils (tonsilloliths, 9.2%), lymph nodes (often due to prior granulomatous infections), thyroid cartilage (9.8%), and vascular structures such as the carotid artery (atherosclerotic calcification, 5.8%) and other rare types (0.1-0.8%) like calcified lymph nodes, antroliths, rhinoliths, phleboliths, and osteoma cutis [4].

Additionally, in a separate study of 160 patients with thyroid enlargement, calcifications were present in 21.5% of cases, manifesting in nodular, flat, curvilinear, cloudy, or mixed patterns. Both benign and malignant goiters may develop these calcifications, which are most often the consequence of previous hemorrhage, necrosis, or epithelial degeneration [5].

On the other hand, malignant calcifications in the neck, like those that are present in thyroid carcinomas, chondrosarcomas, or metastatic lymph nodes, frequently exhibit progressive symptoms like discomfort, rapidly growing masses, or compressive consequences like hoarseness and dysphagia [6-8]. On imaging, these lesions tend to show irregular, coarse, or stippled calcifications within soft tissue masses, often accompanied by infiltration of adjacent structures or enhancing nodal disease. Malignant lesions usually exhibit ill-defined margins, heterogeneous enhancement, and radiologic characteristics suggestive of aggressive behavior, in contrast to benign calcifications, which are stable and strongly marginated [9].

Understanding the anatomical complexity of the neck is essential for the correct interpretation of calcific lesions. The larynx, which is situated anteriorly in the neck between the C3 and C7 vertebrae, is essential for breathing, phonation, and protecting the airway. It is made up of cartilages that are joined by ligaments and membranes, such as the thyroid, cricoid, arytenoid, and epiglottis [10].

The deep neck spaces, separated by fascial planes, include the retropharyngeal, pretracheal, parapharyngeal, visceral, and danger spaces, and are clinically significant for the spread of infections, tumors, and other pathologies [11]. The retropharyngeal space lies posterior to the pharynx and extends to the mediastinum [12]; the pretracheal space houses the thyroid, trachea, and esophagus; and the parapharyngeal space is located laterally beside the pharynx. The carotid space contains major vessels and cranial nerves, while the danger space allows potential spread of infection from the skull base to the diaphragm. These spaces are interconnected and influence the localization, progression, and imaging appearance of neck diseases [13].

This unique case represents a rare and intriguing presentation of deep neck calcification, initially raising concern for a malignant process such as chondrosarcoma. Its unexpected benign nature underscores the diagnostic complexity and the importance of considering atypical yet non-neoplastic entities in the differential diagnosis of neck masses.

## Case presentation

**Figure 1:**
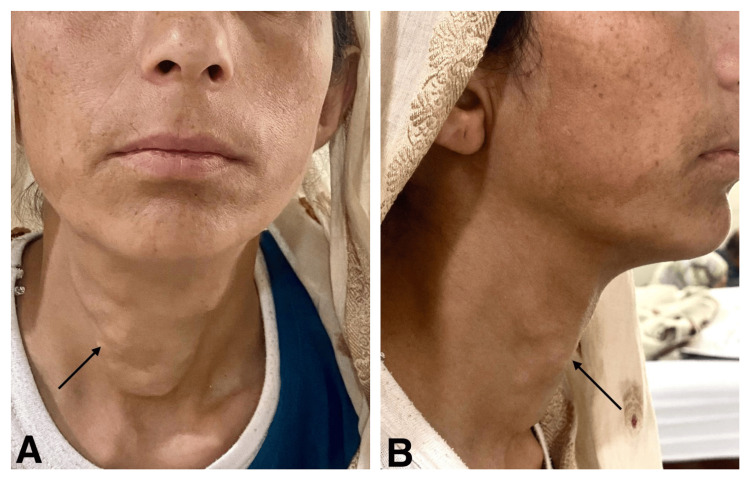
Hard, firm, and fixed swelling on the anterior neck with irregular margins on examination A: Front view. B: Side view Black arrows show hard, firm and fixed swelling on anterior neck with irregular margins.

A 38-year-old female with no known comorbidities presented with complaints of painful, hard, and irregular neck swelling four years back, which was sudden in onset and did not change in size from its initial manifestation. The patient did not complain of any hoarseness or change in voice, dysphagia, or any difficulty in breathing. Examination revealed an approximately 3x4 cm hard, firm, and fixed swelling on the anterior neck with irregular margins, moving with deglutition and tongue protrusion (Figure 1). There was no cervical lymphadenopathy.

Indirect laryngoscopic examination was unremarkable, with normal bilateral vocal cord mobility and an adequate glottic chink; the base of the tongue, epiglottis, bilateral arytenoid cartilages, and pyriform fossae were all normal.

**Figure 2:**
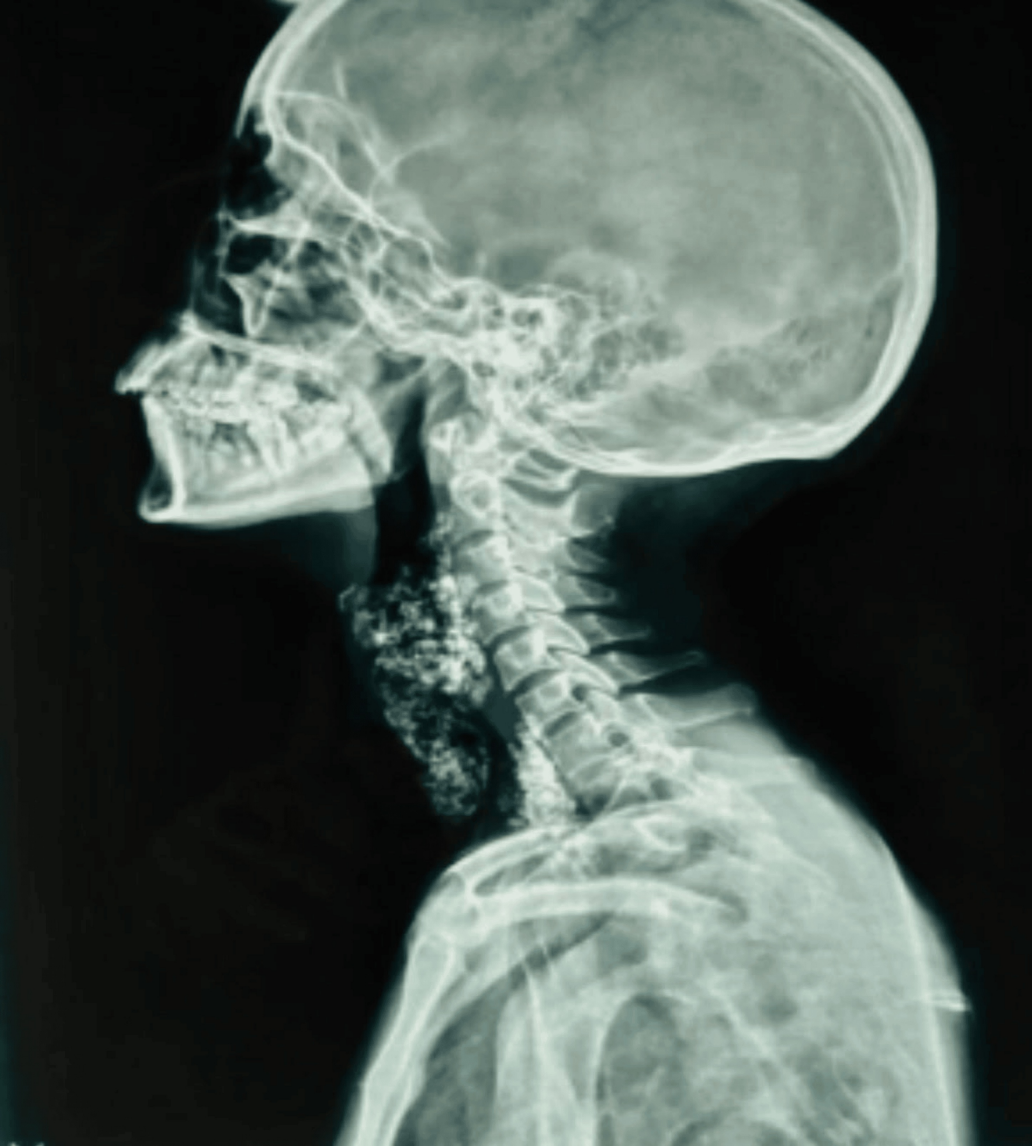
X-ray lateral view of the neck showing diffuse pleomorphic calcifications in the pretracheal, infrahyoid location, extending from C2 to T2 and beyond the thoracic inlet

Neck X-ray radiography showed diffuse pleomorphic calcifications in the pretracheal, infrahyoid location extending from C2 to T2 and beyond the thoracic inlet. The trachea was normal with no pressure effects, as shown in Figure 2. Thyroid function tests showed TSH, T3, and T4 within normal range.

**Figure 3:**
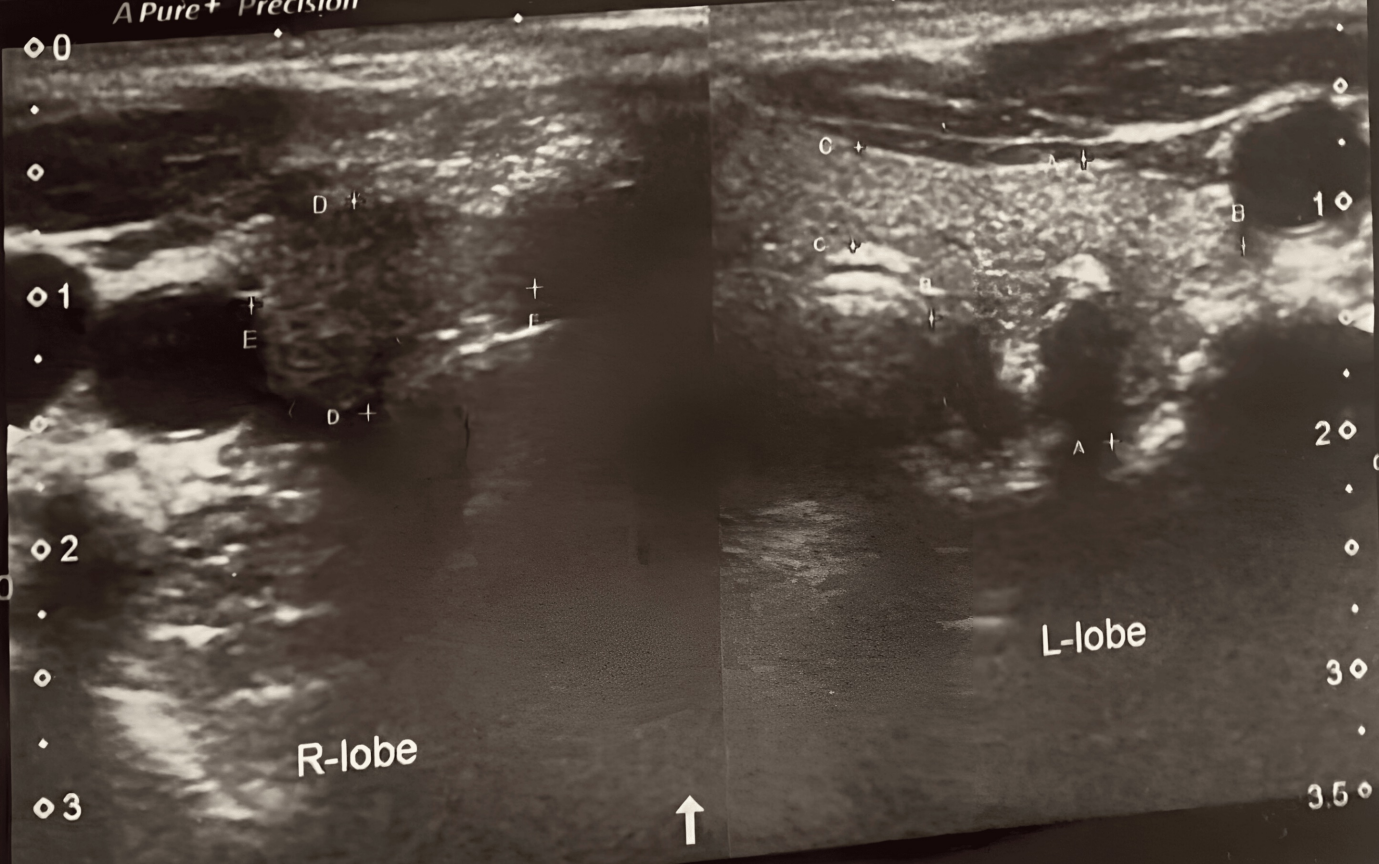
Ultrasound scan revealing a hypoechoic area in the right lobe of the thyroid gland, causing distortion of its margins extending up to the isthmus

Ultrasound scan showed a hypoechoic area in the right lobe of the thyroid gland causing distortion of its margins, extending up to the isthmus. Increased calcification was seen in the thyroid gland (Figure 3).

**Figure 4:**
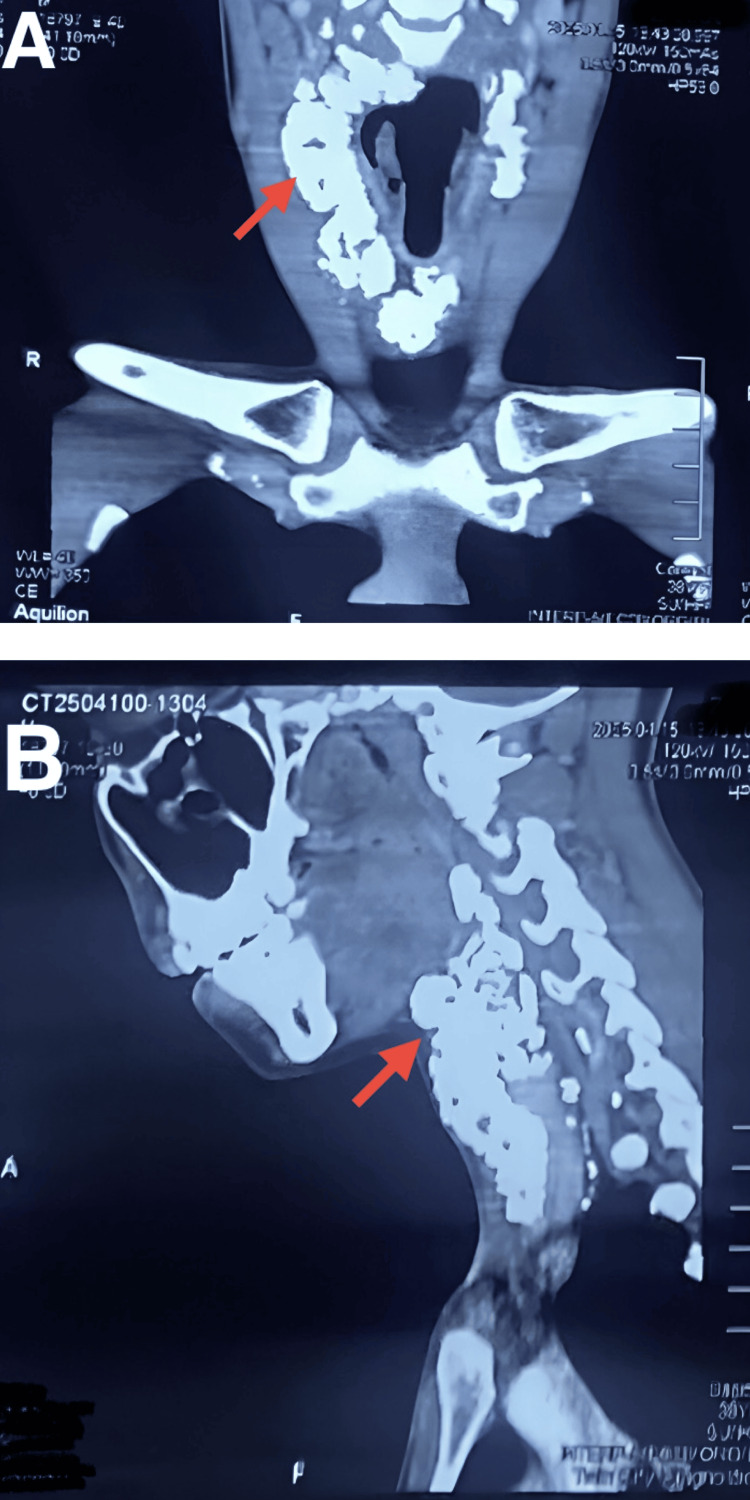
Computed tomography of the neck without contrast A: Coronal view. B: Sagittal view. Red arrows show calcifications.

Computed tomography of the neck with contrast enhancement showed dense calcifications, serpiginous in places, appearing transcompartmental and transfascial in distribution, bilateral in extension, and seen in retropharyngeal, pre-, para-, and retrotracheal positions, as well as para- and retroesophageal (more toward the left side) distribution. Mild effacement of the right vallecula was observed due to retropharyngeal extension. Mild mass effect on the trachea was also noted from the left side due to retrotracheal extension. An 8.9 cm craniocaudal extension was observed from the C7 to T2 level, as shown in Figure 4.

USG-guided FNAC was unsatisfactory and showed hemorrhagic aspirate with calcification.

**Figure 5:**
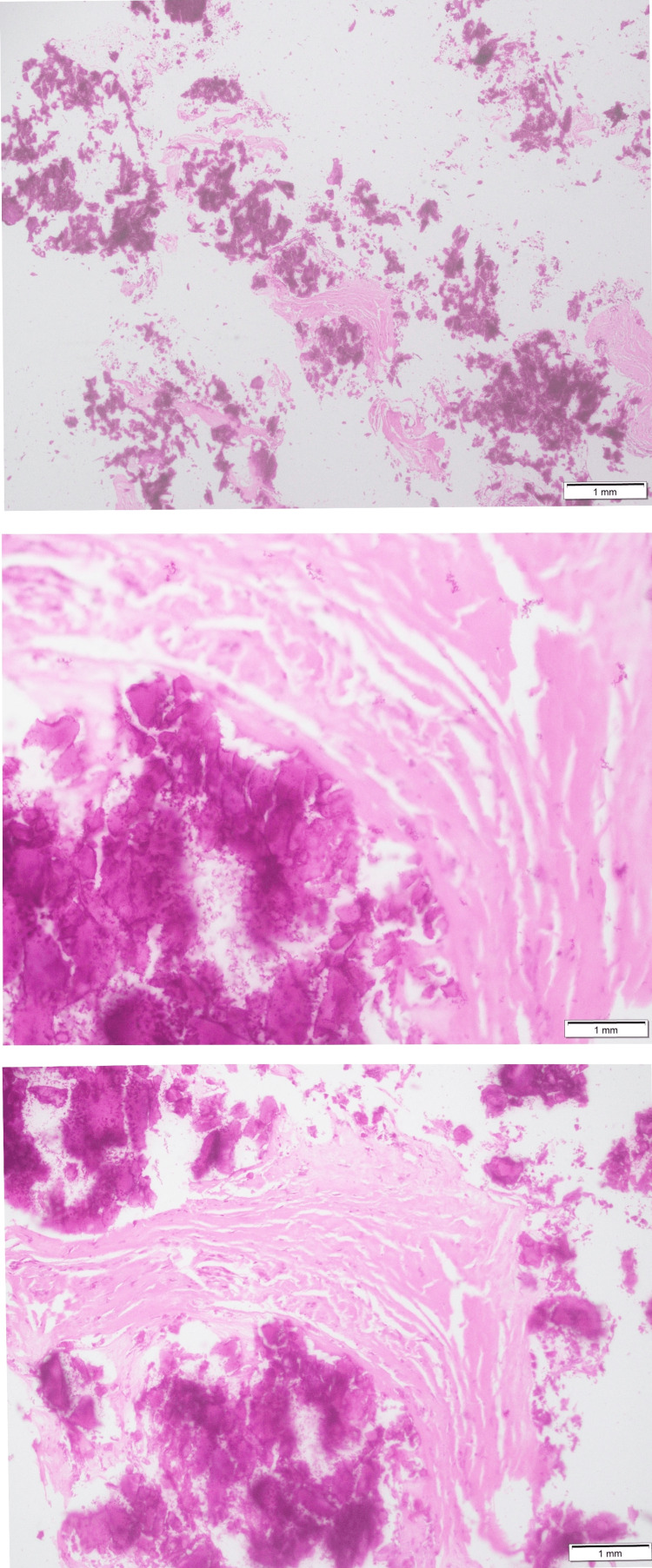
Histopathological image of an incisional biopsy specimen Findings: Calcified tissue and a few fibrous tissue fragments.

The patient underwent incisional biopsy. Gross examination of the specimen showed multiple bony tissue fragments collectively measuring 10×8×2 mm. Microscopic examination predominantly showed calcified tissue and a few fibrous tissue fragments, as shown in Figure 5. No evidence of malignancy or any neoplastic etiology was seen.

As the histopathology report came out negative for any malignancy and the patient also had no compressive signs or symptoms, she was kept on regular long-term follow-up and was counselled about seeking medical advice immediately in case of development of any alarming symptoms.

## Discussion

Benign soft tissue calcification of the neck is an uncommon clinical condition that can present notable diagnostic challenges, primarily due to its ability to clinically and radiologically resemble malignant or aggressive lesions. This case study focuses on a 38-year-old woman in otherwise good health who presented with a firm, painful anterior neck swelling that had been present for four years. The mass had a sudden onset and demonstrated no change in size since its initial manifestation, adding to the complexity of distinguishing it from more sinister pathologies.

Upon clinical examination, our patient’s swelling exhibited movement with both deglutition and tongue protrusion, indicating a potential association with thyroidal tissue or midline embryological remnants. In a similar case, a 31-year-old man with a persistent, firm, non-tender midline neck tumor that had been there since puberty was described by Okubo et al. While a calcified thyroglossal duct cyst was initially suspected, definitive diagnosis following surgical excision and histopathological evaluation revealed a deep soft-tissue leiomyoma with extensive calcification and no evidence of malignancy [14].

Additionally, Ko et al. reported a very distinct presentation in a 19-year-old male who was apparently healthy but developed a painful, rapidly growing mass on the left side of his neck along with compressive symptoms such as vocal fatigue, dysphagia, and shortness of breath. A calcifying fibrous tumor (CFT) was diagnosed based on histological analysis. Psammomatous bodies, dystrophic calcifications, and abundant hyalinized fibrous tissue were among the tumor's distinctive characteristics [15]. The lesion in our patient, on the other hand, remained asymptomatic for a number of years without changing in size or having any clinical significance, emphasizing the broad range of appearance and behavior among benign calcified soft tissue masses of the neck.

A case reported by Hoffman et al. in 2020 involved a young woman who presented with a slow-growing, painless, firm neck mass. Imaging revealed a well-circumscribed, heavily calcified lesion, and histopathology confirmed the diagnosis of calcifying fibrous pseudotumor (CFPT). The lesion was composed of dense hyalinized collagen with psammomatous and dystrophic calcifications and scattered bland spindle cells. The mass exhibited no malignant features, and the patient remained asymptomatic post-excision, with no recurrence on follow-up [16].

In comparison, our case also involved a young female with a hard, irregular anterior neck swelling that remained unchanged over four years. Radiological findings showed extensive, serpiginous, and transcompartmental calcifications, more diffuse than the localized lesion in Sharma et al.’s case. Histologically, our lesion revealed calcified and fibrous tissue without cellular components or neoplastic features. Both cases shared benign pathology and absence of compressive symptoms, supporting conservative or minimal surgical management with close follow-up.

In a case described by Katsuyama et al., a 39-year-old man had a mobile painless neck lump that was first identified by imaging and biopsy as well-differentiated liposarcoma. Histology showed an uncommon benign chondroid lipoma after surgical excision, highlighting how often this condition is misdiagnosed as a sarcoma [17]. On the other hand, our case involved a 38-year-old woman who had significant calcification and a persistent, firm anterior neck swelling. Despite its alarming appearance, the lesion remained stable over four years with no compressive symptoms and was ultimately diagnosed as idiopathic dystrophic calcification. Although both cases resembled malignancy, they were very different in terms of appearance, diagnosis, and treatment strategy.

A patient with a persistent, firm, anterior neck mass that was painless and gradually growing was the subject of a case study by Mirfazaelian et al. Thyroid function tests were within normal ranges, and imaging showed thick, coarse calcifications limited to the thyroid area. After surgical removal, FNAC and a later histological assessment verified benign pathology with no indication of cancer. The patient underwent thyroid surgery primarily due to the suspicious appearance and concern for potential progression [18]. In contrast, the 38-year-old female patient in our study had a similar appearance, including dense calcifications seen on imaging, a firm, fixed, painless anterior neck enlargement that had persisted for four years, and normal thyroid function tests. Our patient's calcifications, however, were more widespread and showed a transcompartmental and transfascial spread from C2 to T2, in contrast to the case reported by Mirfazaelian et al. in Iran. Despite the alarming radiological findings, biopsy confirmed benign dystrophic calcification, and the absence of compressive symptoms or malignancy supported a conservative, non-surgical approach in our patient. This contrast underscores how extent of calcification and clinical features can guide differing management strategies even in cases with similar initial presentations.

When assessing calcified neck lesions, malignant causes of calcification should always be taken into account in the differential diagnosis. Unlike the usual presentation of malignant or compressive neck tumors, our patient did not have any systemic symptoms, voice changes, dysphagia, or dyspnea. According to Selma et al., a 75-year-old man who had been experiencing dyspnea for three weeks had a massive calcified thyroid mass crushing his trachea. This was later determined to be grade 2 chondrosarcoma, necessitating radiation and a total laryngectomy [19]. Despite the extensive transcompartmental and transfascial calcific spread in our case, affecting retropharyngeal, retrotracheal, and paraesophageal spaces, the lesion caused only mild mass effect on the vallecula and trachea, with no significant functional compromise.

## Conclusions

This case describes a 38-year-old woman displaying an unusual presentation of a continuous, solid, and uneven bulge at the front of her neck, along with extensive, snake-like calcifications within several cervical fascial layers. The swelling showed a notable non-progressive pattern over four years, with no associated constitutional or compressive symptoms such as dysphagia, dyspnea, or changes in voice. Despite its alarming clinical appearance, which initially raised suspicion of a malignant etiology of the calcification, the absence of progressive symptoms, normal laboratory parameters, and characteristic imaging findings supported a diagnosis of idiopathic or dystrophic calcification of the deep neck spaces.

It is essential for clinicians to recognize this benign entity in the differential diagnosis of fixed anterior neck masses, particularly in younger patients, to avoid unnecessary surgical intervention or extensive workup. Careful clinical examination combined with radiological assessment can effectively distinguish such benign cases from potentially malignant pathologies. Continued follow-up remains important to monitor for any changes that might suggest alternative diagnoses.

## References

[1] Boskey AL (1988). Calcified Tissues: Chemistry and Biochemistry. Calcium in Human Biology.

[2] Seifert G (1997). Heterotopic (extraosseous) calcification (calcinosis). Etiology, pathogenesis and clinical importance (German). Pathologe.

[3] de Faria LL, Babler F, Ferreira LC, de Noronha Junior OA, Marsolla FL, Ferreira DL (2020). Soft tissue calcifications: a pictorial essay. Radiol Bras.

[4] Acikgoz A, Akkemik O (2023). Prevalence and radiographic features of head and neck soft tissue calcifications on digital panoramic radiographs: a retrospective study. Cureus.

[5] Komolafe F (2023). Radiological patterns and significance of thyroid calcification. Clin Radiol.

[6] Darouassi Y, Touati MM, Chihani M, Nadour K, Boussouga M, Ammar H, Bouaity B (2014). Chondrosarcoma metastasis in the thyroid gland: a case report. J Med Case Rep.

[7] Souha K, Sirine A, Omar W (2023). Anaplastic thyroid carcinoma mimicking cervical tuberculosis: a case report. Ear Nose Throat J.

[8] Gozgec E, Tatar A, Ogul H (2023). Unexpected presentation of occult papillary thyroid cancer; retropharyngeal cystic lymph node metastasis mimicking abscess. Ear Nose Throat J.

[9] Wangaryattawanich P, Agarwal M, Rath T (2021). Imaging features of cartilaginous tumors of the head and neck. J Clin Imaging Sci.

[10] Suárez-Quintanilla J, Fernández Cabrera A, Sharma S (2023). Anatomy, Head and Neck: Larynx. https://www.ncbi.nlm.nih.gov/books/NBK538202/.

[11] MacIsaac MF, Rottgers SA (2024). Anatomy, diagnosis, and clinical management of deep neck space infections. FACE.

[12] Mnatsakanian A, Minutello K, Black AC, Bordoni B (2023). Anatomy, Head and Neck, Retropharyngeal Space. InStatPearls [Internet.

[13] Sutcliffe P, Lasrado S (2023). Anatomy, Head and Neck, Deep Cervical Neck Fascia. Biochem Biophys Res Commun.

[14] Minor J, Rizeq M, Wine T (2013). Mummified leiomyoma of the midline anterior neck: case report and literature review. Ear Nose Throat J.

[15] Baumann KB, Orestes MI, Heaton SM, Whiting RE, Wendzel NC, Foss RD (2020). Calcifying fibrous tumor of the neck. Head Neck Pathol.

[16] Hoffmann H, Beaver ME, Maillard AA (2000). Calcifying fibrous pseudotumor of the neck. Arch Pathol Lab Med.

[17] Katsuyama Y, Shirai T, Terauchi R, Tsuchida S, Mizoshiri N, Mori Y, Kubo T (2018). Chondroid lipoma of the neck: a case report. BMC Res Notes.

[18] Mirfazaelian H, Khademi B, Daneshbod Y (2013). A hard place: calcified neck mass. Am J Med.

[19] Düzcü SE, Coşgun Z, Astarci HM (2021). Laryngeal chondrosarcoma of the thyroid cartilage. Turk Patoloji Derg.

